# Requirements for the selective degradation of CD4 receptor molecules by the human immunodeficiency virus type 1 Vpu protein in the endoplasmic reticulum

**DOI:** 10.1186/1742-4690-4-75

**Published:** 2007-10-15

**Authors:** Julie Binette, Mathieu Dubé, Johanne Mercier, Dalia Halawani, Martin Latterich, Éric A Cohen

**Affiliations:** 1 Laboratory of Human Retrovirology, Institut de Recherches Cliniques de Montréal, 110 Avenue des Pins Ouest, Montreal, Quebec H2W 1R7, Canada; 2 Department of Microbiology and Immunology, Université de Montréal, 2900, Édouard-Montpetit, Montreal, Quebec H3T 1J4, Canada; 3 Department of Anatomy and Cell Biology, McGill University, 3640 University Street Montreal, Quebec H3A 2B2, Canada; 4 Faculty of Pharmacy, Université de Montréal, 2900, Édouard-Montpetit, Montreal, Quebec H3T 1J4, Canada

## Abstract

**Background:**

HIV-1 Vpu targets newly synthesized CD4 receptor for rapid degradation by a process reminiscent of endoplasmic reticulum (ER)-associated protein degradation (ERAD). Vpu is thought to act as an adaptor protein, connecting CD4 to the ubiquitin (Ub)-proteasome degradative system through an interaction with β-TrCP, a component of the SCF^β-TrCP ^E3 Ub ligase complex.

**Results:**

Here, we provide direct evidence indicating that Vpu promotes *trans*-ubiquitination of CD4 through recruitment of SCF^β-TrCP ^in human cells. To examine whether Ub conjugation occurs on the cytosolic tail of CD4, we substituted all four Ub acceptor lysine residues for arginines. Replacement of cytosolic lysine residues reduced but did not prevent Vpu-mediated CD4 degradation and ubiquitination, suggesting that Vpu-mediated CD4 degradation is not entirely dependent on the ubiquitination of cytosolic lysines and as such might also involve ubiquitination of other sites. Cell fractionation studies revealed that Vpu enhanced the levels of ubiquitinated forms of CD4 detected in association with not only the ER membrane but also the cytosol. Interestingly, significant amounts of membrane-associated ubiquitinated CD4 appeared to be fully dislocated since they could be recovered following sodium carbonate salt treatment. Finally, expression of a transdominant negative mutant of the AAA ATPase Cdc48/p97 involved in the extraction of ERAD substrates from the ER membrane inhibited Vpu-mediated CD4 degradation.

**Conclusion:**

Taken together, these results are consistent with a model whereby HIV-1 Vpu targets CD4 for degradation by an ERAD-like process involving most likely poly-ubiquitination of the CD4 cytosolic tail by SCF^β-TrCP ^prior to dislocation of receptor molecules across the ER membrane by a process that depends on the AAA ATPase Cdc48/p97.

## Background

CD4 is a 55-kDa class I integral membrane glycoprotein that serves as the primary co-receptor for human immunodeficiency virus type 1 (HIV-1) entry into cells [1]. CD4 consists of a large lumenal domain, a transmembrane portion, and a 38-residues cytoplasmic tail. It is expressed primarily on the surface of a subset of T lymphocytes that recognizes major histocompatibility complex (MHC) class II-associated peptides and plays a major role in the development and maintenance of the immune system.

Despite the critical role played by CD4 during HIV-1 entry, it is well established that HIV-1 down-regulates cell surface expression of its cognate receptor (reviewed in reference [2]). It is believed that this process prevents superinfection and promotes production of fully infectious virions [3,4]. Down-regulation of CD4 in HIV-1-infected cells is mediated through different independent mechanisms involving the activity of three viral proteins: Nef, Env and Vpu. Early in infection, Nef removes CD4 molecules that are already present at the cell surface by enhancing their endocytosis and subsequent degradation in lysosomes [5]. At later stages of the infection, the envelope precursor gp160, through its high receptor binding affinity and inefficient vesicular transport [6], sequesters newly synthesized CD4 in the endoplasmic reticulum (ER) in the form of Env-CD4 complexes and prevents its transport and maturation to the cell surface [7]. The accessory protein Vpu induces a rapid degradation of newly synthesized CD4 molecules bound to gp160 in the ER [8].

Vpu is an 81-amino acids class I integral membrane protein of 16 kDa that is unique to HIV-1 and simian immunodeficiency virus isolated from chimpanzee (SIVcpz) and a few other monkey species ([9-11] and reviewed in reference [12]). The protein consists of an N-terminal hydrophobic membrane anchor domain of 27 amino acids and a charged C-terminal hydrophilic domain of 54 residues that extends into the cytoplasm [13]. This cytosolic domain contains a highly conserved dodecapeptide sequence encompassing residues 47–58 which comprises a pair of serine residues (S52 and S56) that are phosphorylated by casein kinase II [14,15]. Besides its ability to mediate the rapid degradation of CD4 molecules complexed with Env gp160 in the ER, Vpu was also found to promote efficient release of progeny HIV-1 viruses in different human cell types, including T cells and macrophages, by a mechanism that appears to involve the inactivation of a putative host cell factor that restricts viral particle release in a cell-type dependent manner [10,16-19].

From a mechanistic point of view, HIV-1 Env is not absolutely required for Vpu-mediated CD4 degradation. The role of Env appears to be limited to its ability to retain CD4 in the ER, given that efficient CD4 degradation can be observed in the absence of Env as long as CD4 is retained in the ER through the presence of an ER retention sequence or treatment of cells with Brefeldin A (BFA), a fungal metabolite known to block protein sorting from the ER to the Golgi apparatus [20]. The degradation of CD4 mediated by Vpu involves multiple steps that are initiated by the direct physical binding of Vpu to the cytoplasmic tail of CD4 in the ER [21]. Although the binding of Vpu to CD4 is necessary to induce CD4 degradation, it is not sufficient. Indeed, studies aimed at identifying Vpu partners by two-hybrid screens led to the identification of a host cellular co-factor, β-TrCP, which plays a critical role in Vpu-mediated CD4 degradation by interacting with Vpu in a phosphorylation-dependent manner [22]. The human F-box protein β-TrCP functions as a substrate recognition receptor for the multi-subunit ubiquitin ligase (E3) SCF^β-TrCP ^involved in the ubiquitin (Ub) conjugating pathway (reviewed in reference [23]). The interaction between Vpu and β-TrCP is essential for Vpu-mediated CD4 degradation since substitution mutations of Vpu phospho-acceptor sites, S52 and S56, prevent association with β-TrCP and abolish the effect of Vpu on CD4 turnover [22]. These findings have established a link between the machinery responsible for the ubiquitination of proteins destined for degradation by the proteasome and the enhanced CD4 turnover in presence of Vpu. Indeed, further lines of evidence for an involvement of the Ub-proteasome system in Vpu-mediated CD4 degradation were also reported: 1) Vpu-mediated CD4 degradation is not observed in a mammalian cell line expressing a temperature-sensitive Ub activating enzyme (E1), a key component of the machinery involved in the covalent attachment of Ub to target proteins [24]; 2) over-expression of a mutant Ub (Ub K48/R), which prevents the formation of poly-Ub chains, impairs Vpu-mediated CD4 degradation [24]; 3) Vpu-mediated CD4 degradation is inhibited by specific proteasome inhibitors [24].

Vpu-induced CD4 degradation is reminiscent of ER-associated protein degradation (ERAD), a quality control process in the ER that ensures that only proteins with a native folded conformation leave the organelle for other destinations across the secretory pathway [25]. Misfolded proteins that cannot reach their native state are transferred from the ER to the cytosol by a multi-step process called retro-translocation or dislocation which is thought to involve pore complexes formed by proteins such as Derlin-1 [26,27] or by multi-spanning transmembrane E3 ligases such as Hrd1 [28]. ERAD substrates exposed to the cytosol are acted upon by ER-associated components of the Ub conjugation machinery, extracted from the ER membrane by the AAA ATPase Cdc48/p97 and its associated cofactors Ufd1p and Np14p and degraded by the 26 S proteasome (reviewed in reference [25]). This cellular pathway has been co-opted by some viruses to selectively destroy cellular proteins required for immune defense of the host. For example, two human cytomegalovirus (HCMV) proteins, US2 and US11, are able to target newly synthesized class I MHC (MHC-I) heavy chains (HC) for dislocation from the ER, leading to complete extraction of MHC-I HC from the ER membrane into the cytosol followed by proteasomal destruction [29,30]. Most ERAD substrates are poly-ubiquitinated while undergoing dislocation although the details of recognition, timing and post-translational modification of dislocation substrates can vary depending on the substrates [31-33].

Although part of the molecular machinery that is recruited by Vpu to target CD4 for degradation is reasonably well defined, several aspects of Vpu-mediated CD4 degradation still remain unclear. In particular, direct evidence of CD4 ubiquitination in presence of Vpu in human cells has not been demonstrated. Furthermore, it is unclear whether ER-associated CD4 encounters the cytoplasmic proteasome by a process involving dislocation of CD4 molecules across the ER membrane as described for ERAD substrates. Finally, the role of CD4 ubiquitination in processes underlying Vpu-mediated CD4 degradation remains to be specified. Meusser and Sommer have reconstituted the process of Vpu-mediated CD4 degradation in *Saccharomyces cerevisiae *by expressing human CD4 together with Vpu and human β-TrCP and have provided evidence suggesting that Vpu-mediated proteolysis strictly relies on ubiquitination of CD4 at cytosolic lysine residues prior to export of receptor molecules from the ER membrane [34].

In this study, we have analyzed the process of Vpu-mediated CD4 degradation in human cells. The data presented here provide evidence suggesting that Vpu promotes ubiquitination of CD4 cytosolic tail by SCF^β-TrCP ^and mediates dislocation of the viral receptor across the ER membrane in human cells by a process that might depend on the AAA ATPase Cdc48/p97. Interestingly, in contrast to previous results, Vpu-mediated CD4 degradation and ubiquitination were not found to be entirely dependent on cytosolic lysine residues, raising the possibility that ubiquitination at sites other than lysines might also be involved.

## Results

### Poly-ubiquitination of CD4 is required for Vpu-mediated CD4 degradation

**Figure 1:**
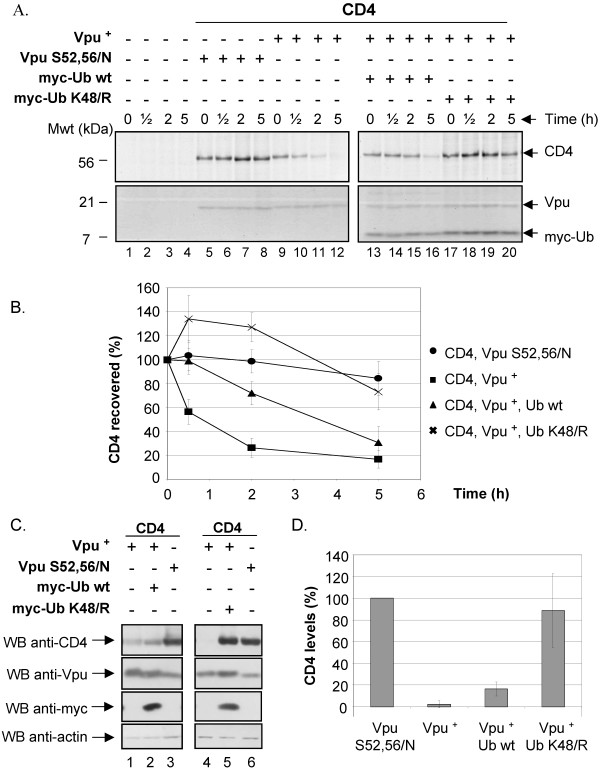
**Poly-ubiquitination of CD4 is required for Vpu-mediated CD4 degradation**. A. HEK 293T cells were mock-transfected or co-transfected with 1.5 μg of SVCMV CD4 wt and 8 μg of SVCMV Vpu^+ ^(Vpu^+^) or the phosphorylation-defective Vpu mutant SVCMV Vpu S52,56/N (Vpu S52,56/N). In parallel, CD4/Vpu transfectants were co-transfected with 8 μg of plasmids encoding his(6)/c-myc-Ub wt (myc-Ub wt) or the TDN mutant of ubiquitin his(6)/c-myc-Ub K48/R (myc-Ub K48/R). Transfected cells were treated with BFA, pulse-labeled with [^35^S]methionine and [^35^S]cysteine and chased with complete media for the indicated time intervals. Cells were then lysed and immunoprecipitated sequentially with anti-CD4 monoclonal and polyclonal antibodies first and then with anti-Vpu and anti-myc antibodies. B. Using quantitative scanning of CD4 bands from three independent experiments, the percentage of CD4 remaining over time as compared to time 0 is plotted for each transfection. C. HEK 293T cells were mock-transfected or co-transfected as described in A. Cell transfectants were treated for two hours with BFA prior to lysis. Steady state levels of CD4, actin and tagged ubiquitin were analysed by western-blot. D. Quantitative analysis from three independent experiments showing the level of CD4 relative to CD4 expressed with Vpu S52,56/N (arbitrarily set at 100%) for each transfectant.

In order to study processes involved in Vpu-mediated CD4 degradation, we established a transient expression system whereby CD4 and Vpu are expressed *in trans *in SV40-transformed human embryonic kidney fibroblasts (HEK 293T) cells. CD4- and Vpu-expressing cells were treated with BFA in order to retain CD4 in the ER before and during metabolic labeling. Pulse-chase radio-labeling analysis followed by immunoprecipitation with anti-CD4 antibodies was performed to ensure that CD4 was specifically degraded by Vpu in this system. Fig. 1 reveals that CD4 turnover was significantly accelerated in presence of Vpu. Furthermore, the effect of Vpu on CD4 was specific since expression of a phospho-acceptor sites mutant, Vpu S52,56/N, which is unable to interact with the E3 Ub ligase complex SCF^β-TrCP ^[22] did not mediate CD4 degradation (Fig. 1A, compare lanes 5–8 with lanes 9–12 and Fig. 1B). Moreover, as previously reported [24,35], addition of specific proteasome inhibitor, such as MG-132, to HEK 293T cells expressing CD4 and Vpu inhibited Vpu-mediated CD4 degradation (data not shown).

We also tested whether poly-ubiquitination of CD4 was required for Vpu-mediated CD4 degradation in HEK 293T cells. For this purpose, we co-expressed CD4 and Vpu with a N-terminal his(6)/c-myc tagged form of wild-type (wt) Ub or a N-terminal his(6)/c-myc tagged form of a transdominant negative (TDN) mutant of Ub, Ub K48/R, that is unable to form poly-Ub chains required for proteasomal degradation [36]. This Ub mutant acts as a chain terminator in the process of poly-ubiquitination since the Ub-acceptor lysine residue at position 48 is mutated for an arginine. In agreement with previous reported data [24], results of Fig. 1A (compare lanes 9–12 with lanes 17–20) and B reveal that expression of tagged-Ub K48/R markedly reduced the rate of Vpu-mediated CD4 degradation, thus suggesting that poly-ubiquitination of CD4 via K48 linkage of Ub moieties was required for Vpu-mediated CD4 degradation. Although expression of wt tagged-Ub had some attenuating effect on Vpu-mediated CD4 degradation (compare lanes 13–16 with lanes 9–12 and Fig. 1B), it was clearly less pronounced than with the TDN tagged-Ub K48/R mutant. In that regard, wt tagged Ub has been previously reported to decrease the rate of degradation of some substrate by the Ub-proteasome system given that fusion of the his-myc tag at the N-terminal of Ub renders poly-Ub-protein conjugates less recognizable by the proteasome [37]. All of these results were also confirmed by analyzing steady-state levels of CD4 by western-blot in Vpu-expressing HEK 293T cells (Fig. 1C and 1D). Overall, these results provide evidence that this expression system in HEK 293T cells supports an efficient degradation of CD4 that is Vpu-specific, depends on the recruitment of β-TrCP, necessitates an active proteasome and requires poly-ubiquitination of CD4.

### Vpu induces ubiquitination of CD4 molecules

**Figure 2:**
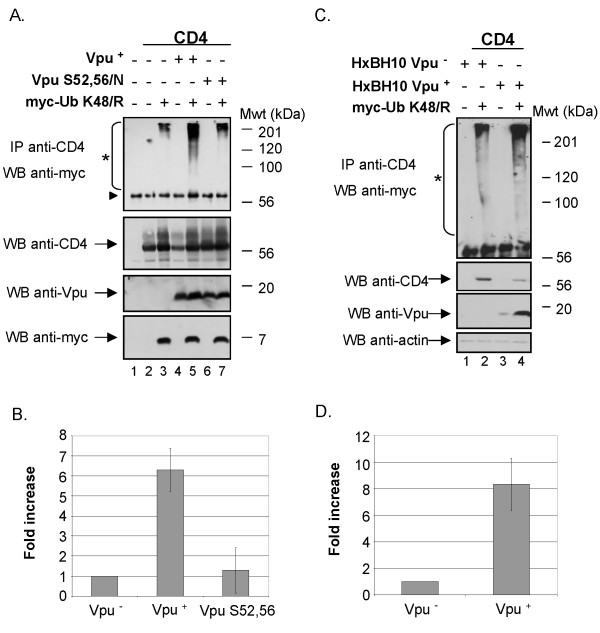
**Effect of Vpu on CD4 ubiquitination**. A. Vpu-mediated ubiquitination of CD4 wt when CD4 is retained in the ER through treatment with BFA. HEK 293T cells were mock-transfected or co-transfected with 1 μg of SVCMV CD4 wt, 8 μg of SVCMV Vpu^+ ^or the phosphorylation-defective Vpu mutant SVCMV Vpu S52,56/N and 8 μg of the TDN mutant his(6)/c-myc-Ub K48/R. Samples were then treated as described in the materials and methods section. CD4 molecules were immunoprecipitated with anti-CD4 polyclonal antibodies prior to western-blot analysis with anti-myc monoclonal antibodies. (triangle) indicates the position of the heavy chains of anti-CD4 antibodies. B. Quantitative analysis of ubiquitinated CD4 conjugates. (asterisk) represents the area of the autoradiogram that was used for quantitation of CD4-Ub conjugates. The histogram shows the relative levels of ubiquitinated CD4 conjugates in presence or absence of a functional Vpu. Relative CD4-Ub conjugate levels were evaluated by quantitation of the signal detected in the area delineated on the autoradiogram relative to total CD4 as determined by quantitation of the band detected with the anti-CD4 antibodies on whole cell lysate. The relative level of ubiquitinated CD4 detected in absence of Vpu was arbitrarily set at 1. The data represent results from seven experiments. C. Vpu-mediated ubiquitination of CD4 wt in condition where CD4 is retained in the ER through binding with HIV-1 Env. HEK 293T cells were mock-transfected or co-transfected with 1 μg of pHIV CD4 wt, 10 μg of provirus encoding Vpu^- ^(HxBH10-vpu^-^) or Vpu^+ ^(HxBH10-vpu^+^) and 20 μg of his(6)/c-myc-Ub K48/R. Samples were then treated as in A but in absence of BFA. D. Quantitative analysis showing the relative levels of ubiquitinated CD4 detected in two independent experiments. Relative levels of ubiquitinated CD4 conjugates were determined as described in B.

Having established that over-expression of Ub K48/R inhibited Vpu-mediated CD4 degradation in HEK 293T cells, we investigated whether we could isolate and directly detect ubiquitinated forms of CD4 that are expected to accumulate under these conditions. Towards this goal, we first analyzed CD4 expression at steady state in Vpu/CD4 HEK 293T transfectants in presence or absence of tagged-Ub K48/R (Fig. 2A). In these experiments, transfected cells were treated with BFA during 2 h prior to lysis to retain newly synthesized CD4 in the ER. Fig. 2A reveals that CD4 levels at steady-state were significantly reduced in presence of Vpu (compare lanes 2 and 4). As expected, expression of tagged-Ub K48/R suppressed the effect of Vpu on CD4 and re-established the amounts of CD4 to levels comparable to those detected in absence of Vpu (compare lanes 5 and 2). To detect CD4-Ub conjugates, cell lysates were first immunoprecipitated with anti-CD4 polyclonal antibodies and the resulting CD4-containing immunocomplexes were subsequently analyzed for the presence of CD4-Ub conjugates by western-blot using anti-myc antibodies. Ubiquitinated forms of CD4 were detected as a typical smear in presence of Vpu (lane 5). Although background high molecular weight ubiquitinated forms of CD4 could still be detected in absence of Vpu (lane 3) or in presence of the non-functional Vpu S52,56/N mutant (lane 7), their levels were not as elevated as in presence of wt Vpu (lane 5). Indeed, quantitative analysis revealed that levels of CD4-Ub conjugates were approximately 6-fold higher in presence than in absence of a functional Vpu (Fig. 2B). The detection of a smear of high molecular weight proteins in presence of Vpu is suggestive of poly-ubiquitination of CD4. Poly-ubiquitination is still possible even if Ub K48/R is over-expressed because cells are expressing endogenous wt Ub that can initiate poly-Ub chains before a molecule of Ub K48/R can prematurely terminate the chain.

Finally, we examined whether we could extend this enhancing effect of Vpu on CD4 ubiquitination to a more physiological system where CD4 is retained in the ER through the formation of complexes with Env glycoproteins instead of BFA treatment. In this system, initially described by Willey and co-workers [20], Vpu and Env glycoproteins are co-expressed from a proviral construct while CD4, that is under HIV-1 long terminal repeat control (pHIV CD4) [24], is expressed only in cells expressing Vpu and Env. Results of Fig. 2C and 2D show that even in a system where CD4 is naturally retained in the ER through binding to HIV-1 Env, Vpu expression increases substantially (approximately 8-fold) the level of CD4 molecules undergoing ubiquitination (compare the levels of CD4-Ub conjugates in lanes 4 and 2 (upper panel) relative to their respective CD4 steady state levels (lower panel) and Fig. 2D).

Overall these results indicate that Vpu promotes poly-ubiquitination of CD4 molecules that are targeted for degradation by the proteasome through the recruitment of the SCF^β-TrCP ^E3 Ub ligase.

### Vpu-mediated CD4 degradation and ubiquitination are not strictly dependent on CD4 cytosolic lysines

**Figure 3:**
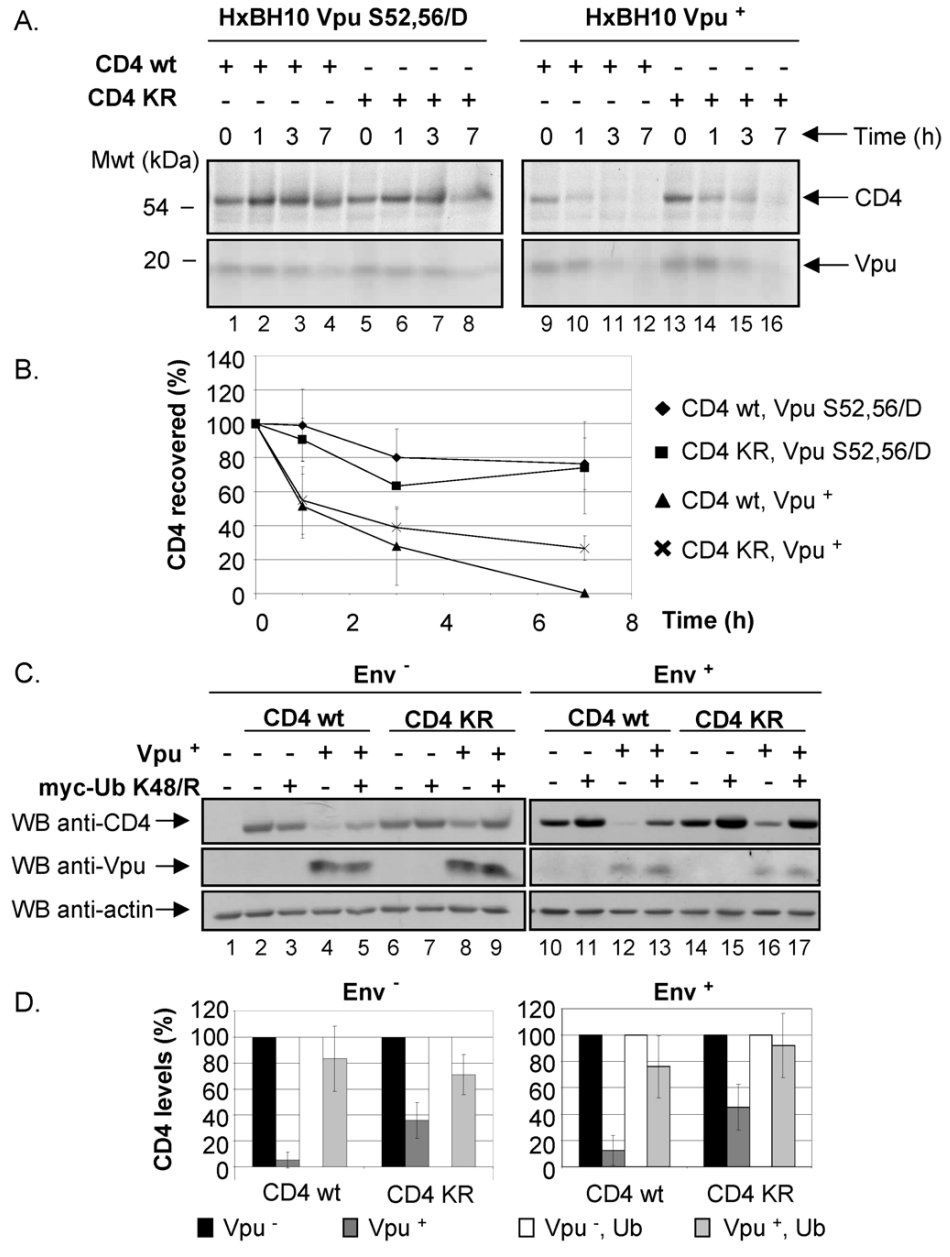
**Effect of Vpu on CD4 molecules lacking lysine residues in the cytoplasmic tail**. A. Analysis of CD4 wt and CD4 KRcyto turnover in presence or absence of functional Vpu by pulse-chase labeling and immunoprecipitation. HEK 293T cells were mock-transfected or co-transfected with 2 μg of pHIV CD4 wt or pHIV CD4 KRcyto and 20 μg of provirus encoding Vpu^+ ^(HxBH10-vpu^+^) or phosphorylation-defective Vpu mutant (HxBH10-vpu S52,56/D). Cells were pulse-labeled with [^35^S]methionine and [^35^S]cysteine and chased in complete medium for the indicated time intervals. Cells were then lysed and immunoprecipitated sequentially with anti-CD4 antibodies first (polyclonal and monoclonal) and then with anti-Vpu antibodies. B. Using quantitative scanning of CD4 bands from two independent experiments, the percentage of CD4 remaining over time as compared to time 0 is plotted for each transfection. C. Effect of Vpu on steady-state CD4 wt and CD4 KRcyto levels. HEK 293T cells were mock-transfected or co-transfected with 1 μg of pHIV CD4 wt or pHIV CD4 KRcyto and 10 μg of proviruses encoding Vpu^- ^or Vpu^+ ^in addition to 25 μg of the his(6)/c-myc-Ub K48/R expressor. In the left panel (Env^-^), a similar experiment was performed except that HEK 293T cells were co-transfected with 10 μg of envelope-defective provirus (HxBc2-pr^-^, vpu^-^, env^- ^or HxBH10-pr^-^, vpu^+^, env^-^) and treated with BFA for 2 h prior to lysis. Cell lysates were then treated as described in the materials and methods section. D. Quantitative analysis of steady-state CD4 levels. CD4 levels in presence of absence of his(6)/c-myc-Ub K48/R were arbitrarily set at 100%. The levels of CD4 in presence of Vpu are shown relative to the corresponding controls. These results are representative of the data obtained in three independent experiments for Env^- ^and five independent experiments for Env^+^.

CD4 contains four potential Ub acceptor lysine residues in its cytoplasmic domain. To determine whether ubiquitination of the cytosolic tail was required for Vpu-mediated CD4 degradation, we analyzed a CD4 mutant, CD4 KRcyto, in which all four cytoplasmic lysine residues were replaced by arginines. The stability of CD4 wt and CD4 KRcyto was first assessed in cells expressing a provirus encoding either wt Vpu (HxBH10-vpu^+^) or Vpu S52,56/D (HxBH10-vpu S52,56/D) as described above in Fig. 2C. Results of Fig. 3A clearly show that both CD4 wt and CD4 KRcyto were unstable in Vpu expressing cells as observed by the decreased recovery of CD4 molecules over the chase period (lanes 9–12 and lanes 13–16). Quantification of CD4 turnover over several experiments indicated an attenuation of the degradation kinetic of CD4 KRcyto as compared to CD4 wt but the protein was clearly susceptible to Vpu-induced degradation (Fig. 3B). In contrast, both CD4 wt and CD4 KRcyto remained stable over the entire 7 h chase period in cells expressing the phosphorylation mutant Vpu S52,56/D (Fig. 3A, lanes 1–4 and lanes 5–8 and Fig. 3B).

Given that previous studies had shown that Vpu-mediated CD4 degradation strictly relied on cytosolic lysine residues in mammalian cells and yeast [24,34], we analyzed the steady-state levels of CD4 wt or CD4 KRcyto in HEK 293T expressing Vpu^+ ^or Vpu^- ^provirus by western-blot. Similar to what we found in pulse-chase experiments, we repeatedly observed a difference in sensitivity to Vpu-mediated degradation between CD4 wt and CD4 KRcyto (Fig. 3C, compare lanes 14 and 16 with lanes 10 and 12 and Fig. 3D, right panel) but clearly, the absence of cytosolic Ub acceptor lysine residues was not entirely preventing the effect of Vpu on CD4 degradation. Similar results were also obtained when steady-state levels of CD4 wt and CD4 KRcyto were analyzed in BFA-treated HEK 293T cells expressing Vpu^+ ^or Vpu^- ^provirus lacking Env (Fig. 3C, compare lanes 2 and 4 with lanes 6 and 8, and Fig. 3D, left panel).

Given that Vpu was reported to interact with the cytoplasmic tail of CD4 in a region (EKKT, residues 416–419 of CD4) that encompasses some of the lysine residues mutated in the CD4 KRcyto mutant (K417, K418), we further tested whether this difference in susceptibility to Vpu-mediated CD4 degradation could be explained by a diminished ability of CD4 KRcyto to associate with Vpu. Binding experiments were performed as described in materials and methods using the Vpu S52,56/N mutant, which binds CD4 as efficiently as Vpu wt but is unable to mediate CD4 degradation [21]. Results from these experiments reveal that CD4 KRcyto associates with Vpu at least as efficiently as CD4 wt, thus ruling-out that the decreased sensitivity of CD4 KRcyto to Vpu-mediated degradation results from reduced Vpu binding efficiency [Additional file 1]. These results were also confirmed by immunoprecipitation of CD4 followed by western-blot using anti-Vpu antibodies (data not shown).

**Figure 4:**
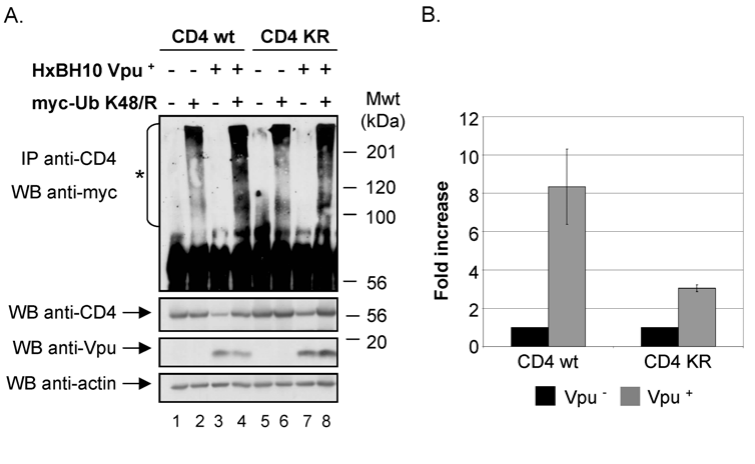
**Effect of Vpu on CD4 KRcyto poly-ubiquitination**. A. HEK 293T cells were mock-transfected or co-transfected with 1 μg of pHIV CD4 wt or pHIV CD4 KRcyto, 10 μg of provirus encoding Vpu^- ^(HxBH10-vpu^-^) or Vpu^+ ^(HxBH10-vpu^+^) and 25 μg of the TDN mutant of Ub his(6)/c-myc-Ub K48/R. Transfected cells were not treated with BFA prior to lysis. Samples were then treated as described in the materials and methods. B. Quantitative analysis of the relative levels of ubiquitinated CD4 conjugates for CD4 wt and CD4 KRcyto in two independent experiments. (asterisk) represents the area of the autoradiogram that was used for the quantitation of CD4-Ub conjugates. Relative levels of ubiquitinated CD4 conjugates were determined as described in Fig. 2B.

Given that CD4 KRcyto was still susceptible to Vpu-mediated degradation, we next evaluated whether CD4 KRcyto could undergo ubiquitination in presence of Vpu. To optimize the recovery of CD4-Ub conjugates, Vpu/CD4 or Vpu/CD4 KRcyto HEK 293T transfectants were made to co-express the TDN Ub K48/R mutant. Analysis of CD4-Ub and CD4 KRcyto-Ub conjugates levels in presence or absence of Vpu was performed as described above for Fig. 2B. Fig. 4A reveals that even though CD4 KRcyto is less susceptible to Vpu-mediated degradation as compared to CD4 wt (compare lanes 1 and 3 with lanes 5 and 7, middle panel), it still undergoes enhanced ubiquitination in presence of Vpu (compare lane 6 and lane 8). However, it is important to note that the relative level of recovered CD4 KRcyto-Ub conjugates was decreased as compared to CD4-Ub conjugates. In fact, quantitative analysis of ubiquitinated CD4 conjugate levels reveals that Vpu enhanced ubiquitination of CD4 KR by approximately 3-fold while it increased ubiquitination of wt CD4 by 8-fold. Altogether, these results suggest that lysine residues in the cytosolic domain of CD4 are not absolutely essential for ubiquitination and degradation of the viral receptor in presence of Vpu. Even-though optimal Vpu-mediated CD4 ubiquitination most probably involves cytosolic lysine residues there must be other sites that are also targeted during Vpu-induced ubiquitination.

### Vpu-mediated CD4 degradation involves the dislocation of ubiquitinated CD4 conjugates across the ER membrane

**Figure 5:**
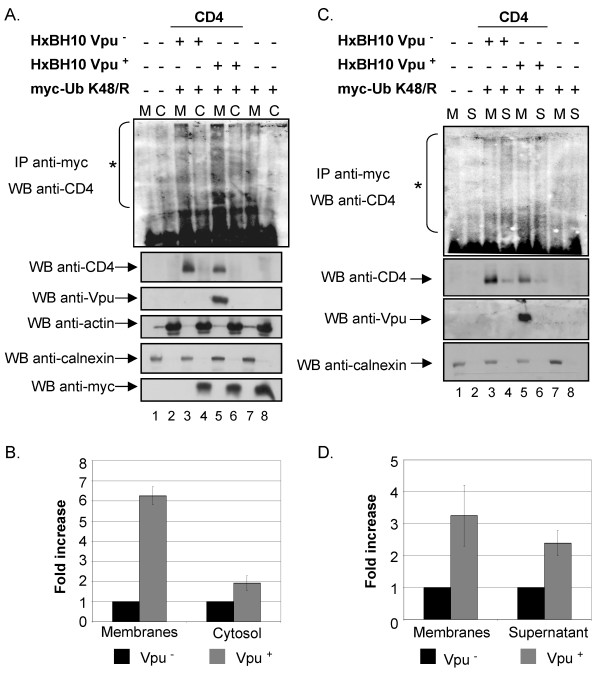
**Vpu-mediated CD4 degradation involves dislocation of ubiquitinated CD4 conjugates from the ER membrane to the cytosol**. HEK 293T cells were mock-transfected or co-transfected with 1 μg of pHIV CD4 wt, 10μg of envelope-defective provirus (HxBc2-pr^-^, vpu^-^, env^- ^or HxBH10-pr^-^, vpu^+^, env^-^) and 15 μg of his(6)/c-myc-Ub K48/R expression plasmid where indicated. Cells were treated with BFA for 2 h before mechanical lysis. CD4-Ub conjugates were immunoprecipitated with anti-myc monoclonal antibodies prior to western-blot analysis with anti-CD4 polyclonal antibodies while control proteins in each fraction were revealed by western-blot. Actin and calnexin were used as cytosolic and membrane controls, respectively. A. Membrane (M) and cytosolic (C) fractions were separated and treated as described in the materials and methods section. B. Quantitative analysis of the relative amounts of ubiquitinated CD4 molecules present in each fraction relative to the amounts measured in absence of Vpu (arbitrarily set at 1). (asterisk) represents the area of the autoradiogram that was used for the quantitation of CD4-Ub conjugates. Non-specific background signal detected in lanes 7 and 8 was subtracted. Relative levels of ubiquitinated CD4 conjugates were determined as described in the legend of Fig. 2B. Error bars reflect standard deviations from duplicate independent experiments. C. Membrane (M) fractions were treated with Na_2_CO_3 _(pH 11) as described in materials and methods. Treated membrane and supernatant (S) were subsequently recovered by centrifugation. Fractions were analyzed as described above in A. D. Quantitative analysis of the relative amounts of ubiquitinated CD4 molecules (as described in the legend of Fig. 2B) present in each fraction relative to the amounts measured in absence of Vpu (arbitrarily set at 1). (asterisk) represents the area that was used for the quantitation of CD4-Ub conjugates. Non-specific background signal detected in lanes 7 and 8 was subtracted. Error bars reflect standard deviations from duplicate independent experiments.

To examine whether CD4 undergoes a process of dislocation across the ER membrane during Vpu-mediated degradation, we conducted subcellular fractionation studies. To optimize recovery and detection of dislocated forms of CD4 targeted for degradation by the cytosolic proteasome, we performed these cell fractionation experiments in conditions where CD4 degradation was inhibited by over-expression of the TDN Ub K48/R mutant. BFA-treated HEK 293T cells expressing CD4/Ub K48/R and Vpu or CD4/Ub K48/R alone were fractionated by mechanical lysis into membrane and cytosolic fractions and each resulting fraction was directly analyzed for the presence of CD4, Vpu and membrane or cytosolic markers, such as calnexin and actin respectively, by western-blot as described in materials and methods. Furthermore, the presence of poly-ubiquitinated forms of CD4 in membrane or cytosolic fractions was determined by immunoprecipitation/western-blot analysis. In contrast to Fig. 2 and 4 and because of technical reasons, ubiquitinated CD4 molecules were detected in these experiments by performing immunoprecipitation using anti-Myc antibodies followed by western-blot using anti-CD4 antibodies. As expected, Vpu and calnexin were detected exclusively in association with membrane fractions (Fig. 5A, lane 5 for Vpu and lanes 1, 3, 5 and 7 for calnexin) whereas actin (lanes 2, 4, 6, 8) or Ub (lanes 4, 6, 8) were recovered in a very large proportion in the cytosolic fractions, thus demonstrating that the fractionation procedure was almost free of membrane or cytosolic contaminations. CD4 molecules were found in the membrane fraction in presence or absence of Vpu (lanes 3 and 5). We could repeatedly recover and detect CD4-Ub conjugates, represented as a smear signal, predominantly in the membrane fraction but also in the cytosolic fraction in absence and in presence of Vpu (Fig. 5A); in some instances, depending on the experiments, we also detected discrete high molecular bands in addition to the smear signal [lane 5 of Additional file 2A and lane 6 of Additional file 2B]. Interestingly, the absolute signal associated with membrane and cytosolic fractions was always more intense in presence than in absence of Vpu (Fig. 5A, compare lanes 3, 5 and 7 as well as lanes 4, 6 and 8, upper panel). The specific levels of CD4-Ub conjugates associated with membrane and cytosolic fractions in absence and in presence of Vpu were calculated relative to the amount of CD4 detected directly by western-blot. As shown in Fig. 5B, quantitative analysis revealed that in presence of Vpu there was approximately a six-fold increase in membrane-associated CD4-Ub conjugate levels relative to the negative control without Vpu (Vpu^-^); in the cytosolic fractions, the levels of CD4-Ub conjugates detected in presence of Vpu were approximately two-fold higher relative to the Vpu^- ^control (Fig. 5B).

To determine whether membrane-associated CD4-Ub conjugates represent CD4 molecules that are still embedded in the membrane while undergoing dislocation or if some of these conjugates are fully dislocated but stay tethered to the cytosolic face of the membrane, we treated membrane fractions with 100 mM sodium carbonate at basic pH (pH 11) (Fig. 5C) and analyzed the treated membrane and resulting supernatant for the presence of CD4-Ub conjugates as described in Fig. 5A. Salt-wash at basic pH (Na_2_CO_3_) but not at neutral pH (NaCl) was previously shown to remove peripheral proteins that are associated with membranes [38]. In this experiment, Vpu (Fig. 5C, lane 5) and calnexin (lanes 1, 3, 5 and 7) were exclusively recovered in the membrane fractions after Na_2_CO_3 _treatment, thus confirming that the integrity of microsomes was maintained during the procedure. Surprisingly, we repeatedly detected small amounts of CD4 in the salt-wash supernatant (lanes 4 and 6, WB anti-CD4 panel) that perhaps represent population of CD4 molecules that are dislocated prior to ubiquitination. Quantitative analysis of the relative CD4-Ub conjugates signal associated with membrane and supernatant fractions revealed that approximately 50% of the membrane-associated signal could be salt washed at basic pH (Fig. 5C, compare lane 3 to lane 4 and lane 5 to lane 6), thus indicating that part of the membrane-associated CD4-Ub signal represents dislocated ubiquitinated forms of CD4 that are associated with the cytosolic face of the membrane. Importantly, in presence of Vpu we detected a 2-3-fold increase in the relative levels of CD4-Ub conjugates associated with the treated membrane fraction and salt-washed supernatant (Fig. 5D). As expected, control experiments where membranes were washed with sodium chloride at neutral pH (pH 7) did not lead to any recovery of CD4-Ub in the supernatant [Additional file 2A]. Conversely, treatment of membranes with RIPA-DOC lysis buffer solubilized CD4-Ub conjugates, which were detected almost completely in the supernatant [Additional file 2B]. As expected, in both conditions the absolute levels of detected CD4-Ub conjugates was more elevated in presence than in absence of Vpu. Overall, these results suggest that Vpu targets CD4 for cytosolic proteasomal degradation by enhancing dislocation of receptor molecules across the ER membrane.

### Expression of a transdominant negative mutant of p97 inhibits Vpu-mediated CD4 degradation

**Figure 6:**
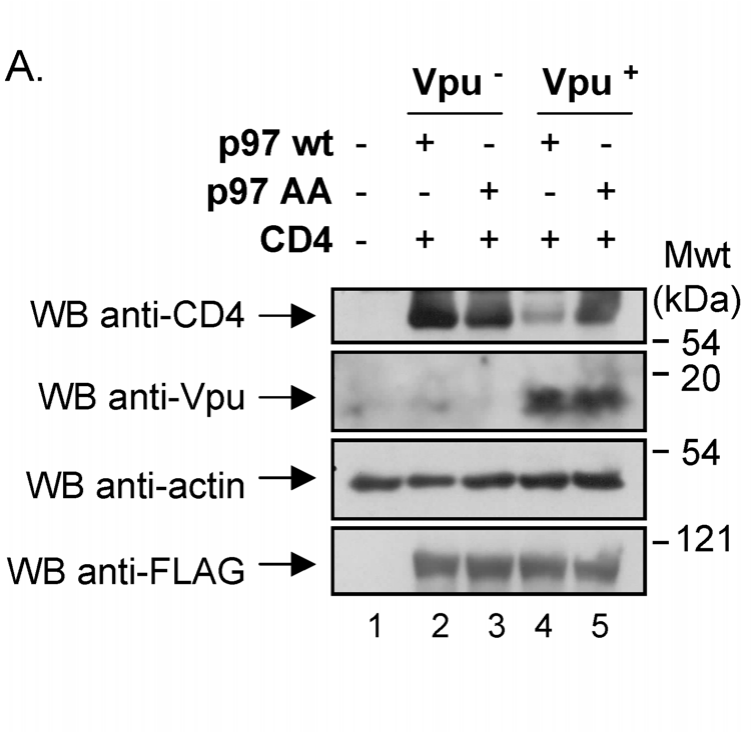
**Effect of a TDN mutant of p97 on Vpu-mediated CD4degradation**. HEK 293T cells were mock-transfected or co-transfected with 1.5 μg of SVCMV CD4 wt, 12 μg of SVCMV Vpu^- ^or Vpu^+ ^and 1 μg of an expression plasmid encoding a FLAG-tagged version of p97 wt or the TDN mutant p97 AA. Cells were treated with BFA for 2 h prior to lysis. Cell lysates were then analyzed by western-blot as described in materials and methods. These results are representative of the data obtained in two independent experiments.

To further confirm that Vpu-mediated CD4 degradation involves a dislocation step, we examined the implication of the Cdc48/p97 ATPase in this process. Mammalian p97 plays an important role in dislocation of ERAD substrates presumably by binding poly-ubiquitinated substrates in conjunction with its cofactors, including Ufd1 and Npl4 [39], and mediating a process of extraction that is energy-dependent [40]. The p97 protein has two ATPase domains and mutants affected in their ability to bind or hydrolyze ATP are no longer able to perform their function in retro-translocation [41]. We took advantage of a well-described p97 TDN ATP binding mutant (p97 AA) [41] and tested its effect on Vpu-mediated CD4 degradation. HEK 293T cells were co-transfected with expression plasmids encoding CD4, Vpu and FLAG-tagged p97 wt or FLAG-tagged p97 TDN mutant and the levels of CD4 were analyzed at steady-state by western-blot. As shown in Fig. 6, expression of the p97 TDN mutant strongly inhibited Vpu-mediated CD4 degradation while wt p97 had no significant inhibitory effect on Vpu ability to degrade CD4 (compare lanes 3 and 5 with lanes 2 and 4). These results were also confirmed by pulse-chase labeling experiments where CD4 turnover was evaluated in presence of Vpu and the p97 TDN mutant or wt p97 (data not shown). Since p97 is directly involved in the dislocation of several ERAD substrates, these results provide additional evidence suggesting that Vpu targets the CD4 receptor for cytosolic proteasomal degradation by a process that involves a dislocation step across the ER membrane.

## Discussion

In the present study, we have conducted a detailed analysis of processes involved in the ER-associated degradation of CD4 receptor molecules induced by the HIV-1 Vpu accessory protein in human cells. Using a TDN mutant of Ub, Ub K48/R, which acts as a poly-Ub chain terminator, we have confirmed previous findings [24] suggesting that poly-ubiquitination of CD4 is required for Vpu-mediated CD4 degradation (Fig. 1). Based on these observations, we attempted to directly detect ubiquitinated forms of CD4, which are expected to accumulate under conditions where Ub K48/R is over-expressed. A similar approach was successfully used to facilitate the isolation and detection of substrates of the Ub pathway such as APOBEC3G in presence of HIV-1 Vif [42]. Under these conditions, we could demonstrate an increased accumulation of high molecular weight CD4-Ub conjugates, typical of poly-ubiquitinated protein targets, in presence of Vpu (Fig. 2). Direct detection of ubiquitinated forms of CD4 in presence of Vpu was achieved both in conditions where CD4 retention in the ER was produced through short treatment of cells with BFA or through formation of Env/CD4 complexes, thus demonstrating that both systems could be used to analyze Vpu-mediated CD4 degradation. Some high molecular weight ubiquitinated CD4 conjugates could be detected in absence or presence of a non functional Vpu mutant unable to recruit the SCF^β-TrCP ^E3 ligase complex, except that their levels were significantly lower than those found in presence of Vpu. It is likely that ubiquitinated CD4 conjugates detected at steady-state in absence of Vpu or in presence of inactive Vpu represent intermediates resulting from the relatively low but normal degradation of misfolded CD4 molecules that occurs through the ERAD pathway in condition of transient ectopic over-expression. Together, these findings provide direct evidence that Vpu promotes *trans*-ubiquitination of CD4 through recruitment of the SCF^β-TrCP ^complex in human cells.

CD4, as a type 1 integral membrane protein, consists of a 38-amino acid cytosolic domain that contains four lysine residues (amino acid positions: K411, KK417-418, and K428) that could serve as acceptor sites for ubiquitination. Ubiquitin conjugation of lysine residues accessible from the cytosol through recruitment of the specific SCF^β-TrCP ^E3 ligase complex by Vpu may represent a very early step in the process of CD4 degradation and precede the transport of the viral receptor through the ER membrane for proteolytic degradation by the cytosolic proteasome. To investigate the role of cytosolic lysine residues in Vpu-mediated CD4 degradation, we used a CD4 mutant in which all four lysines were replaced by arginine residues. In contrast to earlier observations made in HeLa cells [24], replacement of lysine residues in the CD4 cytoplasmic tail did not strictly prevent CD4 degradation by Vpu in HEK 293T cells. In our conditions, even though we detected a significant difference in the protein turnover (Fig. 3A–B) as well as in the steady-state levels (Fig. 3C–D) of CD4 KRcyto and CD4 wt in presence of Vpu, our data also revealed that CD4 KRcyto was still susceptible to Vpu-mediated CD4 degradation. These results suggest that ubiquitination of the cytosolic tail at lysine acceptor sites by the SCF^β-TrCP ^E3 ligase is not strictly required for Vpu-mediated CD4 degradation and, therefore, does not appear to constitute an essential early signal that triggers CD4 targeting to the cytosolic proteasome. Given that poly-ubiquitination of CD4 appears to be required for Vpu-mediated CD4 degradation (Fig. 1), our findings raise the possibility that ubiquitination may occur at sites other than cytosolic lysines. Consistent with this possibility, CD4 molecules lacking cytosolic lysine Ub acceptor sites (CD4 KRcyto) are still capable of undergoing ubiquitination in presence of Vpu, albeit to levels that are lower than wt CD4 (Fig. 4). One possible explanation for Vpu-mediated ubiquitination of cytosolic lysine-less CD4 is that a partial dislocation of the receptor N-termini to the cytosolic side may be required, so that lysine residues in the lumenal domain of CD4 may be accessible for ubiquitination by the cytosolic ubiquitination machinery recruited by Vpu. In that regard, in the specific case of HCMV US2-induced ERAD of MHC-I HC, the replacement of cytosolic tail lysine residues did not affect MHC-I HC dislocation and degradation while internal lysine residues were found to be required for these processes. These results have raised the possibility that US2 could induce a partial dislocation of part of the heavy chain into the cytosol, resulting in cytosolic deposition of lumenal lysine residues [43]. Although, this possibility cannot be completely excluded at this point for Vpu-mediated CD4 degradation, we believe that this scenario is unlikely since replacement of cytosolic lysine residues led to an attenuation of CD4 degradation and to a substantial decrease of CD4 ubiquitination by the SCF^β-TrCP ^E3 ligase (Fig. 4); these observations suggests that Vpu-mediated CD4 degradation involves most probably ubiquitination of the receptor cytosolic tail.

An alternative explanation for the ubiquitination and degradation of cytosolic tail lysine-less CD4 molecules by Vpu is that ubiquitination may occur via non-lysine residues. Interestingly, recent evidence indicate that the mouse gamma herpesvirus (γ-HSV) mK3 E3 Ub ligase, which targets nascent MHC-I HC for degradation by ERAD, mediates ubiquitination via serine, threonine or lysine on the HC tail, each of which was found to be sufficient to induce rapid degradation of HC [44]. The γ-HSV mK3 E3 Ub ligase was found to have the ability to mediate the formation of ester bonds that covalently linked Ub to serine or threonine in the tail of the HC substrate. Unlike MIR 1 (also called kK3), an E3 ligase of Kaposi's sarcoma-associated herpesvirus that requires cysteine residues to ubiquitinate MHC-I [45], a cysteine residue in the HC tail was not required for HC to be a substrate for mK3-induced ubiquitination and degradation. Interestingly, the CD4 cytoplasmic tail contains three serine, three threonine and four cysteine residues in addition to four lysines that could potentially serve as ubiquitin acceptor sites. Vpu-mediated ubiquitination of CD4 cytosolic tail at serine, threonine, cysteine or lysine ubiquitin acceptor sites would obviate the need for a partial dislocation of CD4 before ubiquitination and would suggest a vectorial exit of CD4 from the ER in presence of Vpu. On the basis of these novel findings, a systematic analysis of the role of cytosolic serine, threonine, cysteine and lysine residues in Vpu-mediated CD4 ubiquitination and degradation is warranted.

Our cell fractionation studies reveal that Vpu promoted dislocation of CD4 across the ER membrane since levels of poly-ubiquitinated CD4 molecules found associated with membrane and cytosolic fractions were found to be significantly increased in presence of Vpu (Fig. 5A–B). Furthermore, larger amounts of membrane-associated CD4-Ub conjugates, which likely represent exported ubiquitinated CD4 intermediates still attached to the cytosolic surface of the membrane, were recovered following salt wash treatment in presence of Vpu (Fig. 5D). These cytosolic- and membrane-associated poly-ubiquitinated CD4 molecules represent very likely substrates for the cytosolic 26 S proteasome. Importantly, the process underlying Vpu-mediated CD4 degradation appears to depend on the AAA ATPase Cdc48/p97 since over-expression of a TDN mutant of p97 inhibits efficiently the degradation of the receptor (Fig. 6). Although we cannot rule-out that the effect of the p97 TDN mutant might be indirect, we believe that this result combined with the subcellular fractionation studies provide evidence consistent with a model whereby Vpu targets CD4 for cytosolic proteasomal degradation by a process involving dislocation of the receptor across the ER membrane.

Our findings contrast in part with observations made by Meusser and Sommer in *S. cerevisiae *where they have reconstituted the process of Vpu-mediated CD4 degradation by expressing human CD4 together with Vpu and human β-TrCP [34]. They found that Vpu-mediated proteolysis of CD4 involved dislocation of ubiquitinated intermediates in the cytosol as we found in the present study. However, in their reconstituted biological system this process relied strictly on prior ubiquitination of CD4 at cytosolic lysine residues. Their findings based on data obtained in yeast display one basic difference compared to our results, which indeed suggest that the process of CD4 degradation mediated by Vpu involves a dislocation of CD4 across the ER membrane that is not entirely dependent on prior ubiquitination of CD4 at cytosolic lysine Ub acceptor sites. Even though cytosolic lysine-less CD4 molecules displayed a substantial reduction of Vpu-mediated ubiquitination, they were still substrate susceptible to Vpu-mediated degradation. This apparent discrepancy may reflect differences between human cells and yeast where indeed the CD4 receptor is not normally expressed. Indeed, human CD4, which is a stable protein when expressed in mammalian cells, was found to be rapidly degraded in yeast in the absence of Vpu. On the other hand, we cannot rule-out that Vpu-mediated degradation and ubiquitination of cytosolic lysine-less CD4 may indeed represent a forced pathway used by substrate having no available lysine residues in the cytoplasmic tail. Nevertheless, both studies provide evidence suggesting that Vpu-mediated ubiquitination of the CD4 cytosolic tail represents an early signal triggering dislocation of receptor molecules across the ER membrane for proteolysis by the cytosolic proteasome.

The recruitment of an E3 ubiquitin ligase complex by Vpu that is distinct from those used in classical ERAD raises the possibility that Vpu might target CD4 to a distinct ERAD pathway. Interestingly, three major pathways of ERAD are now emerging, including ERAD-C, ERAD-L and more recently ERAD-M [46-48]. These pathways, which were mostly characterized in *S. cerevisiae*, are involved in the degradation of substrates that display misfolded cytosolic, lumenal or transmembrane domains, respectively. Even though these pathways have been found to involve ER-associated dislocation and ubiquitination machineries of different protein composition, they all appear to rely on the presence of the Cdc48/p97 ATPase complex [49]. It is believed that the ERAD-M and ERAD-C pathways involve dislocation of substrate membrane-anchored portion to the cytosol before the lumenal domain whereas in the ERAD-L pathway the lumenal domain of the misfolded substrate is dislocated first through a channel before the membrane-anchored portion is released in the cytosol. The situation is thought to be similar in mammalian cells but less is known about the different protein complexes involved in the different ERAD pathways. Interestingly, the well-characterized degradation of MHC-I HC by the HCMV proteins US11 and US2 involve different protein complexes and distinct requirements for cytoplasmic lysine residues for dislocation, ubiquitination and degradation. Indeed, MHC-I HC degradation by HCMV US11 involves the recruitment of Derlin-1 [26,27] whereas US2 does not need this interaction to mediate degradation of MHC-I HC [26]. The specific ERAD pathway recruited by Vpu to target the CD4 receptor for degradation by the proteasome remains to be identified. More studies in this area will not only shed light on the molecular mechanism underlying Vpu-mediated CD4 degradation but will also enhance our understanding of ER-associated protein quality control pathway in mammalian cells.

## Conclusion

Our data provide evidence supporting a model whereby HIV-1 Vpu targets CD4 to the ubiquitin-proteasome degradative machinery by a process involving most likely poly-ubiquitination of the CD4 cytosolic tail by the SCF^β-TrCP ^E3 ligase prior to dislocation of CD4 through the ER membrane. Given that lysine residues in the cytosolic domain of CD4 are not absolutely essential for ubiquitination and degradation of the viral receptor in presence of Vpu, there might be sites, other than lysines, that are also targeted during Vpu-induced CD4 ubiquitination.

## Methods

### DNA constructions

SVCMV CD4 was constructed by inserting a XbaI-XbaI cDNA fragment encoding CD4 into the corresponding sites of the expression vector SVCMV expa as described previously [50]. Plasmid pHIV CD4 KRcyto has already been described [24] and is a kind gift from Dr. Klaus Strebel (NIAID, NIH, Bethesda). The four cytoplasmic lysine residues of CD4 were replaced by arginines in pHIV CD4 KRcyto. pHIV CD4 was constructed by inserting a NheI-BamHI fragment from the CD4 cDNA derived from the pT4B expression plasmid [51] into the corresponding sites of pHIV CD4 KRcyto plasmid, thus creating the wild-type (wt) counterpart of pHIV CD4 KRcyto. The plasmid SVCMV CD4 KRcyto was constructed by subcloning a PCR-generated fragment from pHIV CD4 KRcyto into SVCMV CD4.

The Vpu expression plasmid, SVCMV Vpu has been described previously [52]. SVCMV Vpu S52,56/N expressing the corresponding Vpu substitution mutant was generated by PCR-based site-directed mutagenesis as described previously [53].

HxBH10-vpu^+ ^(LTR-gag^+^, pol^+^, vif^+^, vpr^-^, tat^+^, rev^+^, vpu^+^, env^+^, nef^-^-LTR) and HxBH10-vpu^- ^(LTR-gag^+^, pol^+^, vif^+^, vpr^-^, tat^+^, rev^+^, vpu^-^, env^+^, nef^-^-LTR) are two isogenic infectious molecular clones of HIV-1 that differ only in their ability to express Vpu [16]. The phosphorylation mutant of Vpu, HxBH10-vpu S52,56/D, was created from HxBH10-vpu^+ ^using PCR-based mutagenesis. HxBc2-pr^-^, vpu^-^, env^- ^was obtained by replacing the SalI-BamHI fragment of HxBc2-pr^-^, vpu^- ^[54] with the corresponding fragment from HxBc2-vpu^-^, env^-^fs [55]. HxBH10-pr^-^, vpu^+^, env^- ^was obtained by replacing the SalI-BamHI fragment of HxBH10-pr^-^, vpu^+ ^with the corresponding fragment from HxBH10-vpu^+^, env^-^fs [55].

The expression plasmids pCW7 and pCW8 encoding wt Ub and the Ub K48/R transdominant negative (TDN) mutant respectively, have been described previously [56] and were kindly provided by Dr. Ron Kopito (Department of Biological Sciences, Stanford University, Stanford). They both encode N-terminal his(6)/c-myc tagged forms of yeast Ub, which is almost identical to the mammalian counterpart. The plasmids encoding the FLAG-tagged versions of p97 wt and the TDN mutant p97AA were kindly provided by Dr. Martin Latterich (Faculty of Pharmacy, Université de Montréal, Montreal). The TDN p97AA mutant was described previously [40]. The nucleotide sequence of all plasmids was confirmed by automatic DNA sequencing.

### Cell lines and transfections

SV40-transformed human embryonic kidney fibroblasts (HEK 293T) were obtained from the American Type Culture Collection (ATCC, Rockville, MD) and cultured in Dulbecco's modified Eagle medium (Wisent Inc., Saint-Bruno, QC) supplemented with 5% of fetal bovine serum (FBS) (Wisent Inc.) (DMEM+5%). For transfections, 100-mm petri dishes were seeded with 1 or 2 million cells and cultured overnight in DMEM+5%. Cells were then co-transfected with a mixture of the indicated DNA plasmids by the calcium-phosphate method.

### Antibodies and chemical compounds

The anti-CD4 (OKT4) and anti-myc (9E10) monoclonal antibodies were derived from ascitic fluids of Balb/c mice that were injected with the OKT4 or 9E10 hybridoma respectively. The OKT4 and 9E10 hybridomas were obtained from the ATCC. Rabbit polyclonal anti-CD4 (CD4 H-370) antibodies were purchased from Santa Cruz Biotechnology Inc. (Santa Cruz, CA). Rabbit anti-Vpu serum was raised by immunization of rabbits with a synthetic peptide corresponding to amino acids 73–81 of the HIV-1 BH10 Vpu protein [9]. Rabbit polyclonal anti-actin, anti-calnexin and anti-FLAG (M2) antibodies as well as BFA were obtained from Sigma Chemical Co (Saint-Louis, MO). BFA was stored as a stock solution of 10 mM in ethanol at -20°C. MG-132 was purchased from Peptide International (Louisville, KY) and was stored as a 10 mM stock solution in DMSO at -20°C.

### Metabolic labeling and radio-immunoprecipitation

Pulse-chase analysis of CD4 degradation experiments were all performed 48 hours post-transfection. Transfected cells were starved in methionine-free DMEM+5% in the presence of 10 μM BFA for 30 min before labeling. Cells were then pulse-labeled for 30 min with 800 μCi/ml of [^35^S]methionine and [^35^S]cysteine ([^35^S] Protein Labeling mix, Perkin Elmer, Waltham, MA) and chased in complete DMEM+5% supplemented with 10 μM BFA. At the indicated time periods, radio-labeled cells were lysed in radio-immunoprecipitation assay (RIPA-DOC) buffer (140 mM NaCl, 8 mM Na_2_HPO_4_, 2 mM NaH_2_PO_4_, 1% Nonidet-P40, 0.5% sodium dodecyl sulfate, 1.2 mM deoxycholate (DOC), pH 7.2) supplemented with a cocktail of protease inhibitors (Complete, Roche Diagnostics, Laval, QC). For CD4 degradation experiments where Vpu was expressed from HxBH10 provirus, no BFA was added since CD4 ER retention was achieved through HIV-1 Env glycoproteins.

CD4-Vpu binding experiments were performed 48 hours post-transfection. Transfected cells expressing CD4 and a phosphorylation mutant of Vpu (Vpu S52,56/N) were first starved in methionine-free DMEM+5% for 30 min. Cells were then labeled for 2.5 h with 400 μCi/ml of [^35^S]methionine and [^35^S]cysteine and lysed in CHAPS buffer (50 mM Tris, 5 mM EDTA, 100 mM NaCl, 0.5% CHAPS, pH 7.2) supplemented with a cocktail of protease inhibitors.

Following lysis, labeled protein lysates were sequentially immunoprecipitated with anti-CD4 OKT4 monoclonal antibodies only (for binding experiments) or a mixture of OKT4 and CD4 H-370 antibodies (for CD4 degradation experiments) and subsequently with a rabbit anti-Vpu serum as described previously [57]. When indicated, anti-myc antibodies (9E10) were mixed with anti-Vpu antibodies in order to detect his(6)/c-myc Ub fusion proteins. Immunoprecipitates were resolved on a 12.5% SDS-poly-acrylamide tricine gel and analyzed by autoradiography. Scanning of the autoradiograms was performed on an AGFA Duoscan T1200 scanner. Densitometric analysis of autoradiograms was performed with Image Quant 5.0 from Molecular Dynamics (Sunnyvale, CA).

### Protein analysis by immunoprecipitation and western-blots

All experiments were performed 48 h post-transfections. For experiments using SVCMV expressor plasmids to express CD4 and Vpu proteins or experiments using protease and envelope deficient proviruses (HxBH10 pr^-^, env^-^), cells were pre-treated with 10 μM BFA for 2 h when indicated prior to lysis with 0.5%-1% Nonidet-P40 (10 mM Tris, 250 mM glucose, 1 mM EDTA, 0.5–1% Nonidet-P40, pH 7.6) for 30 min on ice. When HxBH10 was used to express Vpu, no BFA was added to the cells. Cell lysates were obtained after centrifugation at 10,000 g in a microcentrifuge for 30 min at 4°C. A sample of each lysate was run directly on 12.5% SDS-poly-acrylamide tricine gel. For detection of ubiquitinated CD4 conjugates, the remaining portion was immunoprecipitated with anti-CD4 polyclonal antibodies (CD4 H-370) and analyzed on an 8% SDS-poly-acrylamide tricine gel. Proteins were then electro-blotted over-night in a Bio-Rad Trans Blot Cell on a 0.45 μm pore size nitrocellulose membrane (Bio-Rad Laboratories, Mississauga, ON) and specific proteins were revealed by western-blotting using anti-CD4 polyclonal antibodies (1:1,000 dilution), anti-Vpu polyclonal antibodies (1:1,000 dilution), anti-actin polyclonal antibodies (1:1,200 dilution), anti-myc monoclonal antibodies (1:1,500 dilution) or anti-FLAG antibodies (1:2,000 dilution) diluted in PBS containing 0.02% sodium azide (NaN_3_). Ubiquitinated forms of CD4 were revealed by western-blotting the membrane containing the anti-CD4 immunoprecipitates with anti-myc monoclonal antibodies. Bound antibodies were then probed with horse-radish peroxidase-linked anti-rabbit (1:7,000 dilution) or anti-mouse (1:6,000 dilution) antibodies, washed extensively and revealed using a standard enhanced chemiluminescence (ECL) detection system.

### Cell fractionation and salt wash experiment

Forty-eight hours post-transfection, HEK 293T cells were treated with 10 μM BFA for 2 h, washed in cold PBS, resuspended in 500 μl of hypotonic buffer (10 mM Tris, 250 mM glucose, 1 mM EDTA) and incubated on ice for 30 min. Cells were then lysed mechanically with a type B Dounce homogeneizer on ice (70 strokes). Cell lysates were centrifuged twice at 250 g in a microcentrifuge at 4°C for 15 min to eliminate unlysed cells and then centrifuged at 10,000 g for 30 min at 4°C to isolate the membrane fraction. The supernatant (cytosolic fraction) was ultra-centrifuged at 100,000 g at 4°C for 1.5 h to eliminate remaining membrane contaminants and then adjusted with lysis buffer (10 mM Tris, 250 mM glucose, 1 mM EDTA, 4% Nonidet-P40, pH 7.6) to a final concentration of 1% Nonidet-P40. The pellet (membrane fraction) of the 10,000 g centrifugation was washed with the hypotonic buffer 4 times prior to lysis in 1% Nonidet-P40 lysis buffer. After lysis, a sample of each fraction was run directly on 12.5% SDS-poly-acrylamide tricine gel while the remaining portion was immunoprecipitated first with anti-myc 9E10 monoclonal antibodies before analysis on an 8% SDS-poly-acrylamide tricine gel. Analysis of proteins in lysates was performed as described above while detection of ubiquitinated CD4 conjugates was performed by western-blotting using anti-CD4 polyclonal antibodies. Polyclonal anti-calnexin antibodies were diluted 1:7,000.

For the salt wash experiment, the cytosolic fraction was discarded and the membrane fraction was either treated with 100 μl of Na_2_CO_3 _(100 mM, pH 11), NaCl (100 mM) or with RIPA-DOC lysis buffer during 10 min on ice. After treatment, samples were centrifuged at 10,000 g for 30 min at 4°C to isolate the membrane fraction (M) and the supernatant (S) was recovered and adjusted to a final volume of 1 ml with 1% Nonidet-P40 lysis buffer. The remaining membrane fraction was lysed with 1 ml of 1% Nonidet-P40 lysis buffer. Each fraction was then analyzed as described above.

## Competing interests

The author(s) declare that they have no competing interests.

## Authors' contributions

JB designed and performed all the experiments and contributed to the writing of the manuscript. MD provided reagents, participated in the design of some experiments and in the revision of the manuscript. JM participated in the execution of several experiments. DH and ML provided original reagents.  EAC conceived the study, participated to data analysis and contributed to the writing of the manuscript. All authors read and approved the final manuscript.

## Supplementary Material

Additional file 1 Analysis of Vpu binding to CD4 wt or CD4 KRcyto. HEK 293T cells were mock-transfected, co-transfected with 1.5 μg of SVCMV CD4 wt or SVCMV CD4 KRcyto and 12 μg of a plasmid encoding a phosphorylation-defective Vpu mutant (SVCMV Vpu S52,56/N) or with 12 μg of SVCMV Vpu S52.56/N alone. Cells were labeled with [^35^S]methionine and [^35^S]cysteine, lysed and sequentially immunoprecipitated with anti-CD4 OKT4 monoclonal antibodies first to observe bound Vpu and then with anti-Vpu antibodies to recover the unbound Vpu proteins. B. Quantitative analysis of the bands in A showing the percentage of binding of CD4 KRcyto to Vpu S52,56/N relative to CD4 wt (arbitrary set at 100%). Error bars reflect standard deviations from duplicate independent experiments. Click here for file

Additional file 2 Salt-wash experiment controls. HEK 293T cells were mock-transfected or co-transfected with 1 μg of pHIV CD4 wt, 10 μg of envelope-defective provirus (HxBc2-pr^-^, vpu^-^, env^- ^or HxBH10-pr^-^, vpu^+^, env^-^) and 15 μg of his(6)/c-myc-Ub K48/R expression plasmid where indicated. Cells were treated with BFA for 2 h prior to mechanical lysis. Membrane (M) were treated with either NaCl (pH 7.0) (panel A.) or RIPA-DOC (panel B.) as described in materials and methods. The treated membranes (M) and supernatants (S) were subsequently isolated by centrifugation. CD4-Ub conjugates were immunoprecipitated with anti-myc monoclonal antibodies prior to western-blot using anti-CD4 polyclonal antibodies whereas control proteins in each fraction were directly revealed by western-blot. Calnexin was used as a membrane-associated protein control. (asterisk) represents the area of the autoradiogram that we considered as poly-ubiquitinated CD4 molecules. Click here for file
