# Supplementary material for: Requirements for the selective degradation of CD4 receptor molecules by the human immunodeficiency virus type 1 Vpu protein in the endoplasmic reticulum
Source: Retrovirology. 2007 Oct 15;4:75. doi: 10.1186/1742-4690-4-75 (PMC2170451; doi:10.1186/1742-4690-4-75)
Supplement: Additional file 1 — Analysis of Vpu binding to CD4 wt or CD4 KRcyto. HEK 293T cells were mock-transfected, co-transfected with 1.5 μg of SVCMV CD4 wt or SVCMV CD4 KRcyto and 12 μg of a plasmid encoding a phosphorylation-defective Vpu mutant (SVCMV Vpu S52,56/N) or with 12 μg of SVCMV Vpu S52.56/N alone. Cells were labeled with [35S]methionine and [35S]cysteine, lysed and sequentially immunoprecipitated with anti-CD4 OKT4 monoclonal antibodies first to observe bound Vpu and then with anti-Vpu antibodies to recover the unbound Vpu proteins. B. Quantitative analysis of the bands in A showing the percentage of binding of CD4 KRcyto to Vpu S52,56/N relative to CD4 wt (arbitrary set at 100%). Error bars reflect standard deviations from duplicate independent experiments. [file 1742-4690-4-75-S1.pdf]

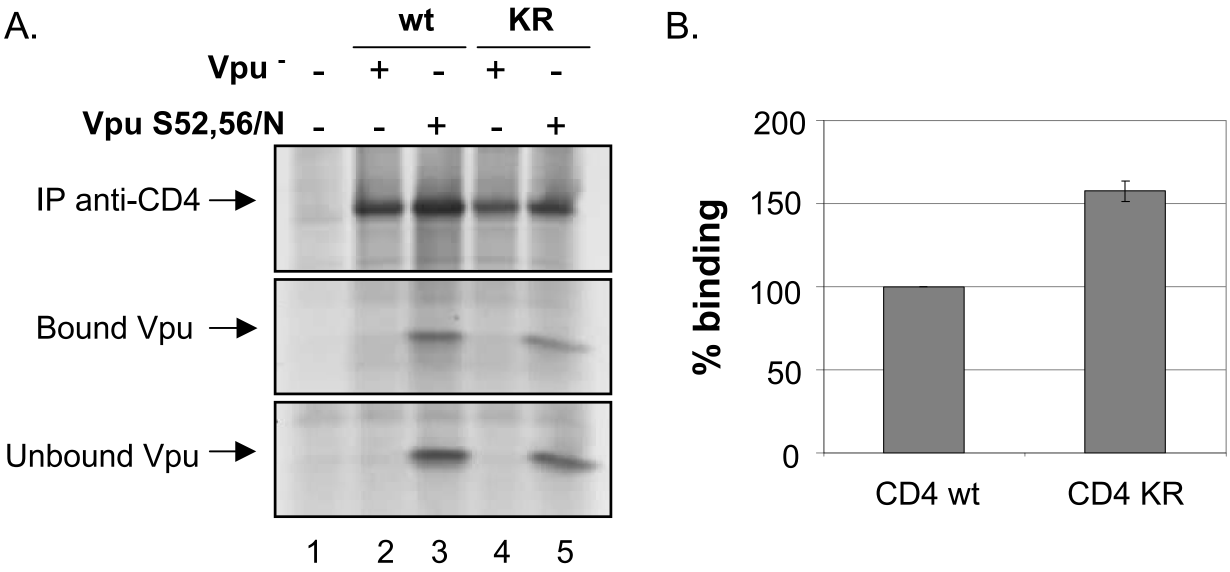

Additional file 1. HEK 293T cells were mock-transfected or co-transfected with 1.5 μg of SVCMV CD4 wt or SVCMV CD4 KRcyto and 12 μg of a plasmid encoding a phosphorylation-defective Vpu mutant (SVCMV Vpu S52,56/N). Cells were labeled with [^{35}S]methionine and [^{35}S]cysteine, lysed and sequentially immunoprecipitated with anti-CD4 OKT4 monoclonal antibodies first to observe bound Vpu and then with anti-Vpu antibodies to recover the unbound Vpu proteins. B. Quantitative analysis of the bands in A showing the percentage of binding of CD4 KRcyto to Vpu S52,56/N relative to CD4 wt (arbitrary set at 100%). Error bars reflect standard deviations from duplicate independent experiments.
