# Supplementary material for: Requirements for the selective degradation of CD4 receptor molecules by the human immunodeficiency virus type 1 Vpu protein in the endoplasmic reticulum
Source: Retrovirology. 2007 Oct 15;4:75. doi: 10.1186/1742-4690-4-75 (PMC2170451; doi:10.1186/1742-4690-4-75)
Supplement: Additional file 2 — Salt-wash experiment controls. HEK 293T cells were mock-transfected or co-transfected with 1 μg of pHIV CD4 wt, 10 μg of envelope-defective provirus (HxBc2-pr-, vpu-, env- or HxBH10-pr-, vpu+, env-) and 15 μg of his(6)/c-myc-Ub K48/R expression plasmid where indicated. Cells were treated with BFA for 2 h prior to mechanical lysis. Membrane (M) were treated with either NaCl (pH 7.0) (panel A.) or RIPA-DOC (panel B.) as described in materials and methods. The treated membranes (M) and supernatants (S) were subsequently isolated by centrifugation. CD4-Ub conjugates were immunoprecipitated with anti-myc monoclonal antibodies prior to western-blot using anti-CD4 polyclonal antibodies whereas control proteins in each fraction were directly revealed by western-blot. Calnexin was used as a membrane-associated protein control. (asterisk) represents the area of the autoradiogram that we considered as poly-ubiquitinated CD4 molecules. [file 1742-4690-4-75-S2.pdf]

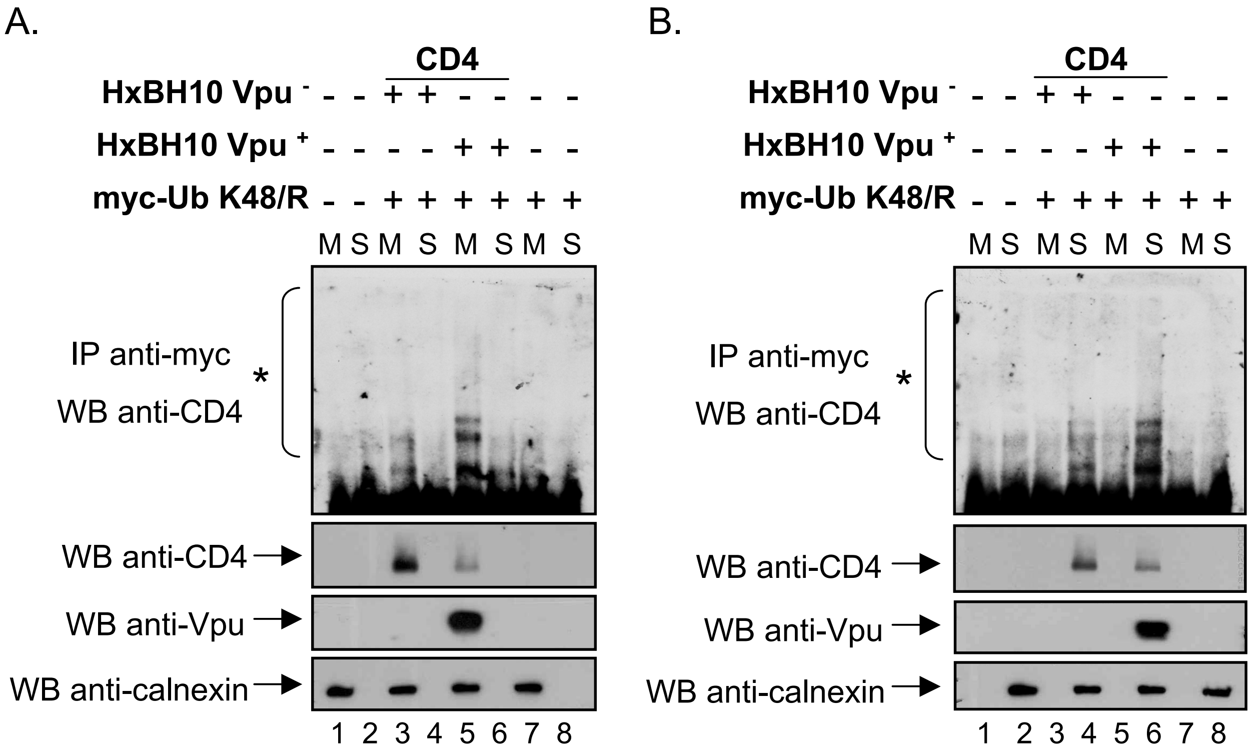

Additional file 2. HEK 293T cells were mock-transfected or co-transfected with 1 μg of pHIV CD4 wt, 10μg of envelope-defective provirus (HxBc2-pr⁻, vpu⁻, env⁻ or HxBH10-pr⁻, vpu⁺, env⁻) and 15 μg of his(6)/c-myc-Ub K48/R expression plasmid where indicated. Cells were treated with BFA for 2 h prior to mechanical lysis. Membrane (M) were treated with either NaCl (pH 7.0) (panel A.) or RIPA-DOC (panel B.) as described in materials and methods. The treated membranes (M) and supernatants (S) were subsequently isolated by centrifugation. CD4-Ub conjugates were immunoprecipitated with anti-myc monoclonal antibodies prior to western-blot using anti-CD4 polyclonal antibodies whereas control proteins in each fraction were directly revealed by western-blot. Calnexin was used as a membrane-associated protein control. (asterisk) represents the area of the autoradiogram that we considered as poly-ubiquitinated CD4 molecules.
